# Worlds Apart – Transcriptome Profiles of Key Oral Microbes in the Periodontal Pocket Compared to Single Laboratory Culture Reflect Synergistic Interactions

**DOI:** 10.3389/fmicb.2018.00124

**Published:** 2018-02-06

**Authors:** Zhi-Luo Deng, Helena Sztajer, Michael Jarek, Sabin Bhuju, Irene Wagner-Döbler

**Affiliations:** ^1^Research Group Microbial Communication, Department of Molecular Infection Biology, Helmholtz Centre for Infection Research, Braunschweig, Germany; ^2^Genome Analytics, Helmholtz Centre for Infection Research, Braunschweig, Germany

**Keywords:** periodontitis, Porphyromonas gingivalis, Treponema denticola, Fusobacterium nucleatum, metatranscriptome, inter-species interaction, SNPs

## Abstract

Periodontitis is a worldwide prevalent oral disease which results from dysbiosis of the periodontal microbiome. Some of the most active microbial players, e.g., *Porphyromonas gingivalis, Treponema denticola*, and *Fusobacterium nucleatum*, have extensively been studied in the laboratory, but it is unclear to which extend these findings can be transferred to *in vivo* conditions. Here we show that the transcriptional profiles of *P. gingivalis, T. denticola*, and *F. nucleatum* in the periodontal niche are distinct from those in single laboratory culture and exhibit functional similarities. GO (gene ontology) term enrichment analysis showed up-regulation of transporters, pathogenicity related traits and hemin/heme uptake mechanisms for all three species *in vivo*. Differential gene expression analysis revealed that cysteine proteases, transporters and hemin/heme-binding proteins were highly up-regulated in the periodontal niche, while genes involved in DNA modification were down-regulated. The data suggest strong interactions between those three species regarding protein degradation, iron up-take, and mobility *in vivo*, explaining their enhanced synergistic pathogenicity. We discovered a strikingly high frequency of Single Nucleotide Polymorphisms (SNPs) *in vivo*. For *F. nucleatum* we discovered a total of 127,729 SNPs in periodontal niche transcripts, which were found in similar frequency in health and disease and covered the entire genome, suggesting continuous evolution in the host. We conclude that metabolic interactions shape gene expression *in vivo.* Great caution is required when inferring pathogenicity of microbes from laboratory data, and microdiversity is an important adaptive trait of natural communities.

## Introduction

Periodontitis is a chronic inflammation of the periodontium leading to destruction of the alveolar bone and finally tooth loss, for which it is the most important reason worldwide (Darveau, 2010). Periodontitis additionally increases the risk for systemic diseases like artherovascular disease, diabetes, rheumatoid arthritis, and certain forms of cancer (Genco and Van Dyke, 2010; Lundberg et al., 2010; Lalla and Papapanou, 2011; Barton, 2017; Michaud et al., 2017). Although periodontitis is the most prevalent infectious disease and dental plaque the most thoroughly studied microbiota of humans, its etiology is still unsolved (Darveau, 2010). Current understanding implies that the periodontal pocket microbiota form a polymicrobial biofilm which continuously interacts with the human host cells, leading to a symbiotic relationship in health; in disease, the microbiota shift to a dysbiotic stage and disrupt host homeostasis by evading immune responses and triggering inflammatory reactions (Kilian et al., 2016; Van der Velden, 2017). Commensal bacteria like *Prevotella nigrescens*, which are present in both health and disease, turn into additional pathogens in the dysbiotic community (Szafranski et al., 2015a).

Three periodontal pathogens are consistently found in periodontal pockets of individuals suffering from chronic periodontitis, namely *Porphyromonas gingivalis, Treponema denticola*, and *Tannerella forsythia* and were named “red complex pathogens” (Socransky et al., 1998). *P. gingivalis*, which is a minor constituent of the periodontal community in health, belongs to the phylum Bacteroidetes and is considered a “keystone pathogen” that can initiate the shift towards a dysbiotic microbial community by evading host defense, triggering an inflammatory response, and inhibiting IL-8 synthesis, which delays the recruitment of neutrophils; as a result, colonization of the periodontium by commensal bacteria is facilitated, additional nutrients become available (degraded protein, hemin/heme) and the community shifts towards dysbiosis (Hajishengallis et al., 2012). Among the traits of *P. gingivalis* important for these mechanisms are gingipains (arginine-specific cysteine proteases) (Imamura, 2003), an atypical lipopolysaccharide which is a potent antagonist of TLR4 (Darveau, 2010), serine phosphatases (SerB) (Hajishengallis et al., 2012; How et al., 2016), peptidyl-arginine deiminase (PPAD) (Maresz et al., 2013) and neuraminidase (Amano et al., 2014).

The second “red-complex” pathogen, *T. denticola*, belongs into the phylum Spirochaetes (Dashper et al., 2011), and can become extremely abundant in periodontitis (up to 50% of the polymicrobial plaque) while it is almost absent in health (Kilian et al., 2016). *T. denticola* is the only motile member of the “red-complex” pathogens and can invade host cells by means of periplasmic flagella. A flagella protein of *T. denticola* was identified as a highly predictive functional biomarker for periodontitis (Szafranski et al., 2015a). *T. denticola* possess many virulence traits, including adhesins, proteases (dentilisin, dentipain), pore forming toxins (dentilisin, cytalysin), proteins for immune activation (Msp, peptidoglycan, lipoprotein) and immune evasion (resistance to defensins, TLR inhibition, Msp) and metal transport (haemin binding protein, lactoferrin binding protein) (Dashper et al., 2011; Visser and Ellen, 2011).

The fact that *P. gingivalis* and *T. denticola* often co-occur in subgingival plaque in periodontitis suggests that they might interact and form a symbiotic relationship. By comparing gene expression of *P. gingivalis* and *T. denticola* in single culture with that in co-culture it was shown that *P. gingivalis* might provide thiamin and glycine to *T. denticola* resulting in improved growth of both (Tan et al., 2014).

*Fusobacterium nucleatum* is one of the most abundant species in the oral cavity in both diseased and healthy individuals (Han, 2015). It is an anaerobic bacterium which has a Gram-negative cell wall and belongs to the phylum Fusobacteria (Bolstad et al., 1996). *F*. *nucleatum* is a key constituent in the periodontal microbiota due to its abundance in periodontal plaque biofilms and its capability to coaggregate with other species (Kapatral et al., 2002; Signat et al., 2011). Animal studies support a causative role of *F. nucleatum* in periodontal infections (Han, 2015). Additionally, *F. nucleatum* is associated with and potentially causative for a wide spectrum of conditions, including adverse pregnancy outcomes, colorectal cancer, inflammatory bowel disease, and cardiovascular disease, and has been isolated from all body sites, including the placenta (Han, 2015). In colorectal cancer, it causes resistance to chemotherapy (Yu et al., 2017). The most important virulence mechanism of *F. nucleatum* mediating such diverse pathogenicities is the adhesin FadA, which binds to cadherins, the cell-junction molecules, and in such a way it can directly invade host cells and the pericellular space (Rubinstein et al., 2013; Han, 2015). We previously showed that in the periodontal pocket, genes for the synthesis of butyrate, a cytotoxic short-chain fatty acid, are expressed by *F. nucleatum* both in health and disease; in chronic periodontitis, however, additional taxa and additional pathways for synthesis of butyrate were recruited (Szafranski et al., 2015a).

Microbiological investigations rely on pure cultures of the species in question which are grown on artificial media at defined cultivation conditions, thus they reveal the potential of a microorganism, but not its actual behavior *in vivo*. In the periodontal pocket of humans, bacteria encounter an entirely different, highly complex environment, where they compete and interact with hundreds of co-occurring bacterial species and are under continuous attack by the immune system of the human host (Kilian et al., 2016). Large differences in gene expression *in vivo* compared to laboratory culture are therefore expected, but until recently, it was technically impossible to exactly determine them. Using next generation sequencing (NGS) the transcriptome of all members of a sample can now be profiled, and such metatranscriptome data can be interrogated for the behaviors of microbes of interest *in vivo*.

The available metatranscriptome studies of the periodontal niche compared gene expression in health and periodontitis. Duran-Pinedo et al. (2014) discovered that commensals expressed virulence factors in disease and identified GO terms associated with disease progression (Yost et al., 2015). Jorth et al. (2014) compared microbial communities in healthy and diseased periodontal pockets in the same individual; they suggested that although the species composition in periodontal pockets varies widely, the metabolic networks operating in disease are conserved. We had previously investigated the taxonomic composition of periodontal pocket bacterial communities in health and disease using 16S rRNA gene sequencing (Szafranski et al., 2015b). The metatranscriptome analysis of those samples resulted in functional biomarkers and showed that *Prevotella nigrescens* turns into an additional pathogen in disease (Szafranski et al., 2015a). Those metatranscriptome data were then analyzed further to identify KEGG pathway enrichment in disease, and to study the activities of Archaea, virus and protozoa as well as the human host (Deng et al., 2017). Here we now focused on transcripts from our three key periodontal pathogens and extracted them from the metatranscriptomes. To obtain an understanding of the response of *P. gingivalis, T. denticola*, and *F. nucleatum* to *in vivo* conditions we compared their gene expression in single culture on laboratory media with that in human periodontal pockets in chronic periodontitis. Our data reveal large similarities in the functional adaptations to *in vivo* conditions for the three pathogens; moreover, they suggest strong interactions between them with respect to protein degradation, iron uptake and mobility, which explain their synergistic pathogenicity. Unexpectedly, we found an enormous microdiversity of all three pathogens *in vivo* in comparison to the laboratory culture of a clonal isolate.

## Materials and Methods

### Bacterial Strains and Cultivation Conditions

The *in vivo* data were derived from periodontal pocket metatranscriptomes of four individuals with periodontitis and 10 without and details regarding sampling and metatranscriptome sequencing have been described in our previous study (Szafranski et al., 2015a). Three single culture RNA-seq datasets from *P. gingivalis* were derived from a study from Hovik et al. (2012) in which *P. gingivalis* strain W83 was cultured in three different media. Those sequencing data contained about 15 million single end reads per sample with a length of 50 bp.

*Treponema denticola* ATCC 35405 was cultivated in DSM medium 909^1^ at 37°C in an anaerobic chamber (Don Whitley Scientific, Shipley, England) which provided an atmosphere of 80% N_2_, 10% H_2_, and 10% CO_2_. For RNA isolation, after 3 h of growth, 5 ml culture was harvested in the log phase, and after 8 h of growth, 5 ml was sampled in the stationary phase, respectively, from two replicate cultures.

*Fusobacterium nucleatum* ATCC 25586 was cultivated in modified (resazurin was omitted, since it interferes with OD measurements; vitamin K1 concentration was 10-fold increased; hemin concentration was 100-fold reduced) DSMZ medium 104^1^ at 37°C in an anaerobic chamber (Don Whitley Scientific, Shipley, England) which provided an atmosphere of 80% N_2_, 10% H_2_, and 10% CO_2_. For RNA isolation, 5 ml culture was withdrawn at log phase (3 h) and stationary phase (7 and 10 h) respectively from two replicate cultures.

### RNA Extraction and Sequencing

Total RNA was isolated from 5 ml of bacterial culture using the RNeasy RNA Isolation Kit (Qiagen, Germany). mRNA enrichment was carried out with the Ribo-Zero rRNA Removal Kit according to the manufacturer’s instructions, using 1,2 μg of total RNA solved in 27 μl of nuclease free water (Qiagen, Germany). Enriched mRNA was further purified by ethanol precipitation and analyzed using capillary gel electrophoresis (Bioanalyser Agilent, Germany) to verify the depletion of 16S rRNA and 23S rRNA. Integrity of RNA was evaluated using a Bioanalyzer 2100 (Agilent, Germany). The mRNA enrichment yielded about 100 ng mRNA in 20 μl of water. Paired-end mRNA-seq strand specific libraries were prepared with the Script Seq Illumina Kit. Illumina HiSeq 2500 Sequencer (Illumina, Germany) was utilized to produce paired-end reads with a length of 110 base pairs.

### Sequencing Data Preprocessing

Primers and sequencing adaptors were removed from raw sequencing data, followed by clipping the bases with a quality score below 20 from the reads to achieve cleaned reads with Fastq-Mcf (Aronesty, 2011). Reads shorter than 50 bp after trimming were eliminated. The cleaned transcriptome sequencing data of *T. denticola* and *F. nucleatum* in single laboratory culture were submitted to the European Nucleotide Archive database (ENA) and with BioProject ID: PRJEB23061. For the single culture transcriptomic data of *P. gingivalis*, which were downloaded from SRA and had a read length of 50 bp, reads shorter than 20 bp after trimming were discarded. Thereafter, the remaining rRNA reads were eliminated by SortMeRNA v2.0 (Kopylova et al., 2012).

### Short Reads Mapping and Extraction of Species-Specific Reads

For determining the expression level of genes *in vivo*, we extracted *P. gingivalis, T. denticola*, and *F*. *nucleatum* reads from the metatranscriptomes (Szafranski et al., 2015a) using Kraken (Wood and Salzberg, 2014) and BBMAP (Bushnell, 2014). Kraken uses the K-mer strategy and the lowest common ancestor (LCA) algorithm to determine the taxon for a given read. The detailed workflow is as follows: first, the metatranscriptomic data were mapped onto a reference database consisting of prokaryote genomes (2786), virus genomes (4418), and the human genome (ver. GRCh38) downloaded from NCBI, in which the genomes of *P. gingivalis, T. denticola*, and *F*. *nucleatum* were included. Subsequently, the reads originating from *P. gingivalis, T. denticola*, and *F*. *nucleatum* based on the taxonomy were retrieved from the metatranscriptome using BBMAP. The extracted reads were then mapped against the corresponding reference genome using BWA with the BWA-MEM (Li, 2013) algorithm followed by read counting for each gene with FeatureCounts (Liao et al., 2014). For paired end reads with 110 bp, a seed length of 31 was applied, while for RNA-seq data (single end 50 bp) of single culture from *P. gingivalis*, the default seed length of 19 was adopted.

### Differential Expression Analysis and GO Term Enrichment Analysis

The differential expression (DE) analysis was performed using edgeR (Robinson et al., 2010). The raw counts of the genes were normalized to make the *in vivo* and laboratory culture data comparable using the trimmed mean of M value (TMM) method which is offered by edgeR. After DE analysis, genes with FDR <=0.05 were considered as significantly differentially expressed. Those significantly up- and down-regulated genes were then used as the gene lists of interests for GO term enrichment analysis performed by topGO (Alexa and Rahnenfuhrer, 2010). Before the enrichment analysis, GO terms were assigned to each gene using UniProt (UniProt Consortium, 2014). As GO terms contain many redundant functions, we reduced the redundancy of the enriched GO terms and visualized the results using REVIGO (Supek et al., 2011).

### Variants Calling

To investigate differences in the presence of variants on the transcriptome level between *in vivo* conditions and laboratory culture, we utilized SAMtools (Li et al., 2009), BCFtools (Narasimhan et al., 2016), VCFtools (Danecek et al., 2011), and BEDtools (Quinlan and Hall, 2010) to accomplish the variants calling based on the reads alignment files (SAM files). All sample files were pooled to identify the variants. The resulting variants were filtered in terms of the read depth, mapping quality and base quality of sequencing to achieve more confident variants (sites with mapping quality <20 or read depth >100 were marked as low-quality variants). The diversity of the SNPs per gene was calculated based on Shannon diversity defined as:

$$D=\overset{n}{\underset{i=1}{\sum }}-p_{i}\log_{2}p_{i}$$

$$p_{i}\,\;=\;\;\frac{Ai}{\Sigma_{i=1}^{n}\,\;\left(A_{i}\right)}$$

where *n* is the number of SNPs in a given gene, *A*_i_ is the frequency of the altered allele at the *i*th SNP locale relative to the reference sequence. Hence *p*_i_ is the probability of the presence of a given SNP among all detected SNPs. For the calculation of the diversity, only the SNP with A/(A+R) ≥ 0.1 were taken into account.

### Statistics

The PCoA was performed using R based on Bray–Curtis dissimilarity. DE analysis was conducted by edgeR with exact test, the *P*-values in the DE analysis were corrected to FDR with the “Benjamin Hochberg” method for multiple comparisons. The genes with FDR of DE analysis ≤0.05 were considered as differentially regulated. GO term enrichment analysis was carried out with Fisher’s exact test based on hypergeometric distribution using the R package topGO. The FDR of each GO term was also calculated and listed in the **Supplementary Table S1**. GO terms with enrichment analysis *P*-value ≤ 0.05 were taken as input for REVIGO.

## Results

### Study Design and Summary of Sequencing Data

The metatranscriptome samples (*in vivo* samples) have been described previously (Deng et al., 2017). They were derived from 14 individuals, four of which had been diagnosed with chronic periodontitis. **Supplementary Figure S1** shows the relative abundance of the three-species studied here in these samples. *F. nucleatum* was present both in health and disease and comprised up to 25% of all reads. By contrast, *T. denticola* and *P. gingivalis* transcripts were barely detectable in health. Therefore, their mRNA reads were only extracted from the four patients with periodontitis. After quality control and rRNA removal we extracted 224,669 ± 321,128 putative mRNA reads per sample (14 samples) for *F. nucleatum*, 269,739 ± 161,109 reads per sample for *P. gingivalis* (four samples), and 72,791 ± 32,740 reads per sample for *T. denticola* (four samples).

For *P. gingivalis* laboratory culture data we utilized a published dataset based on cultivation on three different media (Hovik et al., 2012). *F. nucleatum* and *T. denticola* were cultivated on the media suggested by the culture collection under anaerobic conditions at 37°C. Samples were taken during exponential and stationary phase of growth with two replicas each and mRNA was sequenced as described (see methods for details). After quality control and rRNA removal, 55,385,310 ± 4,605,386 reads per sample were obtained for *F. nucleatum*, (four samples), 40,558,785 ± 6,092,565 for *T. denticola* (six samples), and 8,628,943 ± 1,567,710 for *P. gingivalis* (three samples). Details regarding the sequencing data can be seen in **Supplementary Table S1 Sheets 1, 2**.

### Gene Expression of *P. gingivalis* in Periodontitis Compared to Laboratory Culture

**FIGURE 1:**
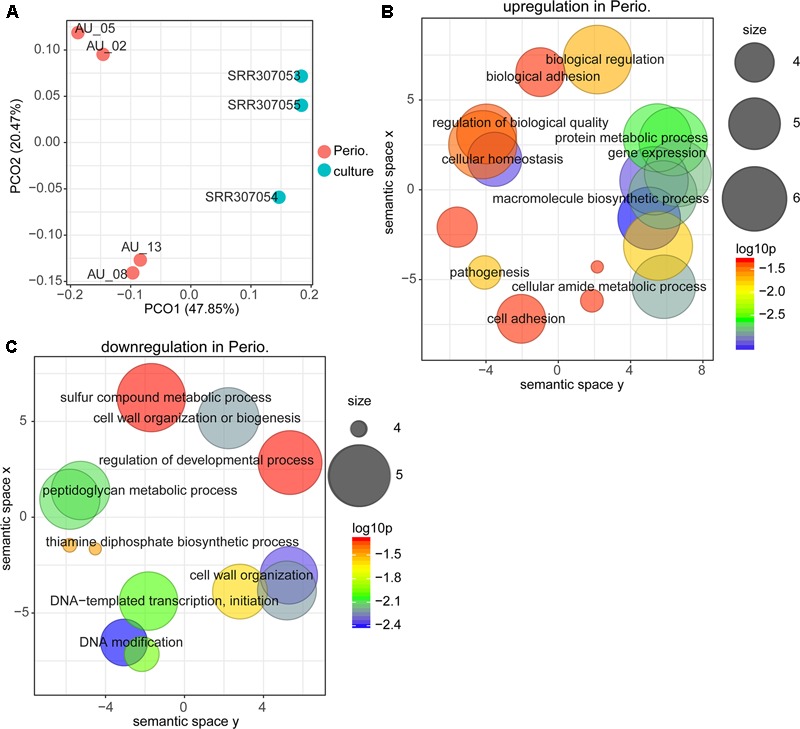
Comparison between gene expression in periodontitis and laboratory culture for *Porphyromonas gingivalis.* **(A)** Principal coordinates analysis (PCoA) of transcriptional profiles from four periodontal pocket samples (chronic periodontitis) and laboratory cultures on three different media. **(B,C)** Gene ontology (GO) terms up-regulated **(B)** and down-regulated **(C)** in periodontitis. All GO terms with enrichment *P*-value ≤ 0.05 were summarized and visualized by REVIGO (Supek et al., 2011), and the size of the bubble indicates the number of merged terms.

PCoA (principal coordinate analysis) showed (**Figure 1A**), that the expression profile of *P. gingivalis in vivo* was completely distinct from that on all three laboratory media. We determined enrichment of GO terms based on differentially expressed genes. Functions involved in protein metabolism, translation, cell adhesion and pathogenesis were more active *in vivo* (**Figure 1B** and **Supplementary Table S1 Sheet 5**), while DNA methylation, thiamine biosynthesis and cell wall organization were up-regulated in laboratory culture (**Figure 1C**).

We then had a closer look at the differentially expressed genes. We observed that 125 genes were significantly up-regulated *in vivo*, whereas 166 were significantly down-regulated (**Supplementary Table S1 Sheet 4** and **Table 1**). A fimbrilin gene and two cysteine protease genes were highly expressed in periodontitis. Among the most prominent upregulation (average fold change of 70) was the *hmu* gene cluster (PG1551-PG1556, *hmuY, hmuR, hmuS, hmuT, hmuU, hmuV*). A large variety of transporters were upregulated *in vivo*, including a multi-antimicrobial extrusion transporter (MATE), numerous ABC transporters and TonB-dependent receptors. The number of genes that were significantly down-regulated was even larger. Among them were genes encoding metabolic enzymes (e.g., glycerol dehydrogenase, glycosyl transferase, phosphoribosyltransferase), DNA modifying enzymes (transposases, integrases, restriction-modification system enzymes) and transcriptional regulators. Intriguingly, the clustered regularly interspaced short palindromic repeats (CRISPR) associated proteins were considerably down-regulated and the most strongly down-regulated gene *in vivo* was the surface protein PgaA.

**Table 1:** Differentially expressed genes (|log2FC| ≥ 5) between periodontitis and laboratory culture in *Porphyromonas gingivalis.*

Genes	log2FC	log2CPM	FDR	Product	Gene
PG1019	7.555157	10.38787	3.62E-21	Putative lipoprotein	
PG1181	7.218641	8.524394	1.04E-14	TetR family transcriptional regulator	–
PG0222	7.084658	6.081433	0.005578	Histone-like family DNA-binding protein	–
PG2133	6.637346	8.215433	2.21E-12	Putative lipoprotein	–
PG1551	6.38076	11.65887	1.36E-18	HmuY protein	hmuY
PG2134	6.343568	8.924579	7.13E-16	Putative lipoprotein	–
PG1552	6.328257	10.56244	4.71E-17	TonB-dependent receptor HmuR	hmuR
PG1554	6.146314	6.953868	4.43E-07	HmuT	hmuT
PG1555	5.922429	6.856549	7.01E-06	HmuU	hmuU
PG1556	6.029691	6.654237	2.54E-07	HmuV	hmuV
PG1858	6.310506	11.24736	3.07E-17	Flavodoxin FldA	–
PG1553	6.011982	10.89276	4.43E-16	CobN/magnesium chelatase, HmuS	hmuS
PG1055	5.721684	10.13226	9.86E-07	Thiol protease	tpr
PG2008	5.571255	11.36346	3.74E-11	TonB-dependent receptor	–
PG1467	5.118896	7.279726	3.46E-08	UbiE/COQ5 family methlytransferase	–
PG2132	5.010408	9.743426	4.43E-07	Fimbrilin	fimA
PG0850	-6.94149	3.985838	0.038459	Excisionase DNA-binding protein	–
PG2135	-6.96172	4.009491	0.037717	Putative lipoprotein	–
PG0225	-7.08273	4.105006	0.030378	ISPg4, transposase	–
PG0019	-7.2357	4.202793	0.020536	ISPg4 transposase	–
PG1515	-7.44897	4.384304	0.012294	Ribulose bisphosphate carboxylase-like protein	–
PG2038	-7.59084	4.510893	0.027908	*N*-acetylmuramoyl-L-alanine amidase	–
PG1514	-7.64219	4.534787	0.008586	Glycerol dehydrogenase	–
PG1436	-7.92391	4.752115	0.003722	ATPase	–
PG1513	-8.29828	5.043848	0.000698	Phosphoribosyltransferase/phosphoglycerate mutase	–
PG1531	-8.99779	5.613199	5.57E-05		
PG0860	-9.05442	5.655394	7.22E-05	Transcriptional regulator	–
PG0838	-10.0629	6.518683	2.79E-07	Integrase	–
PG0971	-10.1306	6.573451	2.48E-07	McrBC restriction endonuclease system, McrB subunit	–
PG0110	-10.4796	6.887122	2.19E-07	Glycosyl transferase	–
PG0544	-10.5353	6.934028	1.49E-08	Type I restriction-modification system, M subunit	–
PG0111	-10.7694	7.142878	9.11E-09	Capsular polysacharride biosynthesis gene	–
PG2016	-11.2452	7.56776	1.37E-09	CRISPR-associated helicase Cas3	cas3
PG0862	-11.6934	7.978136	1.06E-13	Type IIS restriction endonuclease	–
PG0861	-11.7768	8.055359	9.12E-15	Snf2/Rad54 family helicase	–
PG0742	-12.0578	8.317474	1.59E-17	Antigen PgaA	pgaA

We then looked at those genes that were most highly expressed (log2 CPM ≥ 12) both in periodontitis and laboratory culture (**Table 2**). Hemagglutinin protein HagA, HagE, gingipain Kgp/HagD, receptor antigen RagA, and arginine-specific cysteine proteinase prtRII were among the most highly expressed genes under both conditions, confirming their prominent role for the pathogenesis of periodontitis.

**Table 2:** Highly expressed genes (log2CPM ≥ 12) in both periodontitis and laboratory culture in *P. gingivalis.*

Genes	log2FC	log2CPM	FDR	Product	Gene
PG1837	1.004755	14.86717	0.300333	Hemagglutinin protein HagA	–
PG1844	-1.28113	14.73178	0.072097	Kgp/HagD	
PG2024	0.19243	14.64685	0.903392	Hemagglutinin protein HagE	hagE
PG0185	-2.01591	13.53432	0.066922	Receptor antigen ragA protein	ragA
PG0387	-0.09914	13.1116	0.955313	Elongation factor Tu	tuf
PG0506	-0.13087	13.02622	0.943774	Arginine-specific cysteine proteinase	prtRII
PG1940	0.676166	12.66887	0.456598	Elongation factor G	fusA
PG1232	-0.09676	12.63848	0.955313	Glutamate dehydrogenase	gdh
PG1764	-0.61913	12.57631	0.554892	3-oxoacyl-ACP synthase	fabF
PG0395	0.184241	12.43774	0.922275	DNA-directed RNA polymerase subunit beta	rpoC
PG0394	0.313422	12.39457	0.824867	DNA-directed RNA polymerase subunit beta	rpoB
PG0692	-0.92232	12.34672	0.260195	4-hydroxybutyryl-CoA dehydratase	abfD
PG0186	-2.23945	12.21699	0.215431	Lipoprotein RagB	ragB
PG0389	0.952604	12.15031	0.375171	Transcription antitermination protein NusG	nusG

### Gene Expression of *T. denticola* in Periodontitis Compared to Laboratory Culture

**FIGURE 2:**
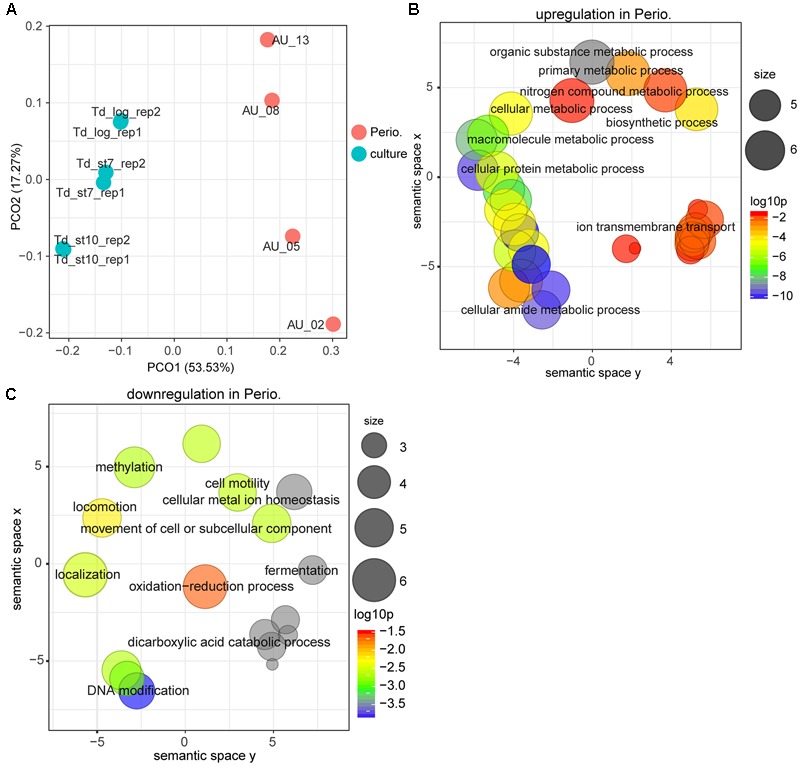
Comparison of the transcriptional profiles between periodontitis and laboratory culture for *Treponema denticola.* **(A)** PCoA of transcriptional profiles from four periodontal pocket samples (chronic periodontitis) and laboratory cultures at log or stationary phase. **(B,C)** GO terms up-regulated **(B)** and down-regulated **(C)** in periodontitis. All GO terms with enrichment *P*-value ≤ 0.05 were summarized and visualized by REVIGO, and the size of the bubble indicates the number of merged terms.

For *T. denticola*, too, the transcriptional profile *in vivo* was massively different from that in laboratory culture (**Figure 2**). The GO term enrichment analysis based on up-regulated or down-regulated genes suggests that gene expression, cell adhesion, protein metabolism and ion transporters were enriched *in vivo*, whereas GO terms of DNA methylation and cell motility were enriched in laboratory culture (**Figures 2A,B** and **Supplementary Table S1 Sheet 8**).

The differential expression analysis identified 257 genes that were up-regulated whereas 730 genes were down-regulated (**Supplementary Table S1 Sheet 7** and **Table 3**). Thus, the number of genes up-regulated in periodontal pocket was two times less than that in laboratory culture, indicating strong selective pressure. As in *P. gingivalis*, ABC transporters, iron uptake transporters and oligopeptide/dipeptide transporter as well as the MATE transporters were the most strongly up-regulated genes in the periodontal pocket. Notably, a pathogen-specific surface antigen of *T. denticola* was considerably up-regulated *in vivo* indicating a strong interaction with the host immune system and with other bacteria. This gene was annotated based on a characterized homologous gene *tpd* in *T. pallidum* encoding Tp34 which is a 34 kDa membrane antigen (Deka et al., 2007). This protein is probably involved in iron acquisition via its propensity to bind lactoferrin (Deka et al., 2007).

**Table 3:** Differentially expressed genes (15 most up- and down-regulated in periodontal pocket) between periodontitis and laboratory culture in *Treponema denticola.*

Genes	log2FC	log2CPM	FDR	Product
2740075	8.578301	6.071104	4.99E-27	Iron compound ABC transporter ATP-binding protein
2740077	8.364144	8.753274	6.49E-40	Iron compound ABC transporter periplasmic iron compound-binding protein
2740076	7.592179	7.353986	4.21E-30	Iron compound ABC transporter permease
2741549	7.115278	8.253119	9.95E-35	MATE family transporter
2740073	6.892528	6.784015	1.36E-20	Oxygen-independent coproporphyrinogen III oxidase
2740845	6.749295	7.81299	5.75E-11	ABC transporter permease
2739036	6.659903	2.914318	1.69E-12	ABC transporter ATP-binding protein
2739143	6.592251	1.053299	1.65E-07	ABC transporter permease
2740800	6.471231	7.951346	8.73E-32	ABC transporter ATP-binding protein/permease
2740799	6.29813	8.534355	3.43E-31	ABC transporter ATP-binding protein/permease
2740388	6.102319	7.656629	1.43E-16	ABC transporter ATP-binding protein/permease
2740074	5.79486	3.974965	4.37E-13	Flavodoxin
2740386	5.79021	4.182447	1.06E-09	ABC transporter ATP-binding protein/permease
2740844	5.704174	8.845092	1.77E-17	ABC transporter ATP-binding protein
2740389	5.658901	6.992283	4.37E-13	ABC transporter ATP-binding protein/permease
2739225	-12.3905	6.430921	4.07E-06	funZ protein
2739011	-12.5252	6.550985	8.43E-07	MarR family transcriptional regulator
2740067	-12.6029	6.620306	9.64E-07	DNA-binding protein
2741609	-12.7715	6.770585	2.62E-07	ABC transporter ATP-binding protein
2740870	-12.7833	6.781314	2.50E-06	Serine/threonine protein phosphatase
2741689	-12.8611	6.850028	1.12E-05	Amino acid permease
2740070	-12.991	6.966664	5.29E-07	Phage integrase family site specific recombinase
2739708	-13.2838	7.228467	2.43E-07	Group 1 glycosyl transferase
2740626	-13.3455	7.28336	1.03E-06	Bacteriocin ABC transporter ATP-binding/permease
2739585	-13.4335	7.362577	7.53E-09	Lipoprotein
2740086	-14.0697	7.936201	1.20E-07	Lipoprotein
2741521	-14.2329	8.084806	2.80E-11	Surface protein
2739407	-14.916	8.712038	5.04E-12	Oligopeptide/dipeptide ABC transporter periplasmic peptide-binding protein
2739558	-15.3404	9.107236	1.35E-08	M20/M25/M40 family peptidase
2739010	-16.2571	9.975078	2.39E-10	Hemolysin

As in *P. gingivalis* the list of down-regulated genes comprised all cellular functions, but was much longer and more diverse. Five type I restriction modification system related genes, one type II restriction endonuclease gene and one CRISPR associated Cas1 protein gene were strongly down-regulated *in vivo* similar as in *P. gingivalis*. Phage related genes were also down-regulated (e.g., phage minor structural protein, phage integrase, phage terminase). The most strongly down-regulated gene *in vivo* was a hemolysin. This down-regulation was caused by the fact that its expression was not detectable *in vivo* using the sequencing depth of the metatranscriptome samples, while its expression in laboratory culture was high.

The most highly expressed genes of *T. denticola* both *in vivo* and in laboratory culture are shown in **Table 4**. Flagellar filament proteins, major outer sheath protein (Msp), glycine reductases and dentilisin were highly expressed under both conditions.

**Table 4:** Highly expressed genes (log2CPM ≥ 12) in both periodontitis and laboratory culture in *T. denticola.*

Genes	log2FC	log2CPM	FDR	Product
2740984	0.24863	15.45597	0.815141	Flagellar filament core protein
2740151	-1.75702	14.2833	0.060708	Major outer sheath protein (Msp)
2739619	-0.22007	14.14716	0.765312	Filament protein A
2739378	-0.87024	14.08339	0.18421	Glycine reductase complex selenoprotein GrdB2
2739379	0.325458	13.28488	0.644964	Glycine reductase complex proprotein GrdE2
2739062	-0.54955	13.11282	0.567464	Oligopeptide/dipeptide ABC transporter periplasmic peptide-binding protein
2740832	0.004274	13.09963	1	Peptide ABC transporter peptide-binding protein OppA
2741592	0.346423	12.6235	0.544976	Flagellar filament core protein
2740833	-0.02755	12.27185	1	Lipoprotein
2738999	-1.87782	12.24685	0.064381	Basic membrane protein
gene773	-1.23962	12.19334	0.261539	Dentilisin
2739736	-0.29963	12.18486	0.649203	Glycine cleavage system T protein
2739631	0.043716	12.10314	0.981134	Malate dehydrogenase

### Gene Expression of *F. nucleatum* in Periodontitis and Health Compared to Laboratory Culture

**FIGURE 3:**
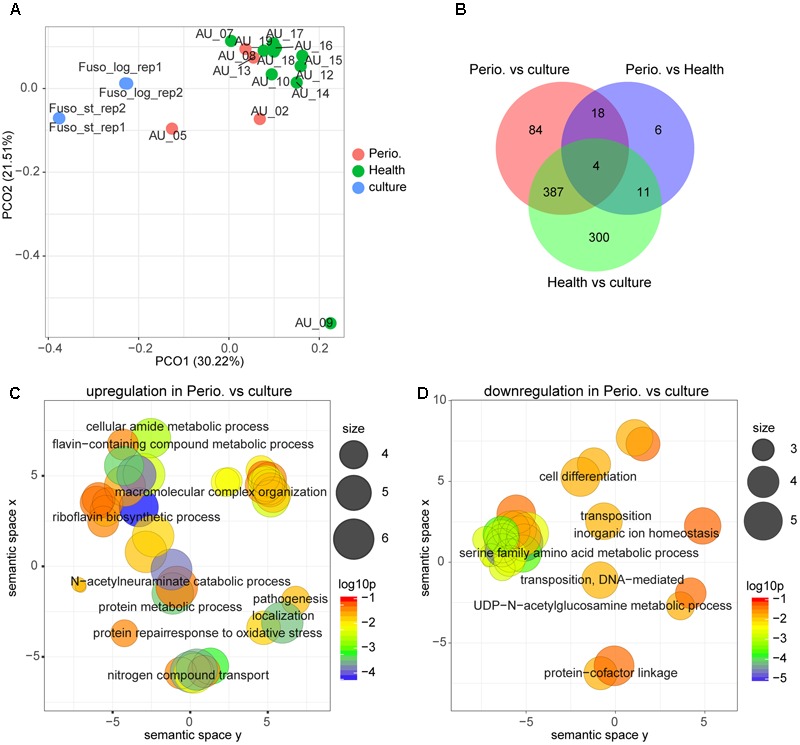
Comparison of the transcriptional profiles between periodontitis, health and laboratory culture for *F. nucleatum*. **(A)** PCoA of transcriptional profiles of *Fusobacterium nucleatum* in four periodontal pocket samples from patients with chronic periodontitis, 10 periodontal pocket samples from healthy individuals and four laboratory cultures at log or stationary phase. **(B)** Venn diagram showing the number of differentially expressed genes in each comparison. **(C,D)** GO terms up-regulated **(C)** and down-regulated **(D)** in periodontitis in comparison to laboratory culture. All GO terms with enrichment *P*-value ≤ 0.05 were summarized and visualized by REVIGO, and the size of the bubble indicates the number of merged terms.

**Figure 3A** shows that the transcriptional profile of *F. nucleatum* was highly diverse. Most *in vivo* transcriptional profiles clustered together and were distinct from those on laboratory media, irrespective of disease status. However, there were two samples of individuals with periodontitis which were intermediate between *in vivo* conditions and those in the laboratory, and one sample from a healthy individual which was completely distinct. In this individual, the abundance of *F. nucleatum* was very low. Accordingly, the overlap between those three conditions was small (**Figure 3B**). By using GO term enrichment analysis, we found that GO terms related to protein metabolism, pathogenesis and transport were up-regulated in periodontitis compared with laboratory culture (**Figure 3C** and **Supplementary Table S1 Sheet 13**). On the other hand, serine family amino acid metabolic process was down-regulated in periodontitis (**Figure 3D**).

When comparing gene expression in periodontitis with laboratory culture, we found 493 differentially expressed genes, of which 143 genes were up-regulated and 350 genes were down-regulated in periodontitis (**Supplementary Table S1 Sheet 10** and **Table 5**). A hemin receptor was the most strongly up-regulated gene. Peptide transporter genes, Na+ driven multidrug efflux pump genes, and cell surface protein genes were highly expressed in periodontitis. On the other hand, numerous genes encoding methyltransferase, two lipopolysaccharide biosynthesis related genes and the gene encoding CRISPR-associated protein Cas5 were down-regulated in periodontitis compared with laboratory culture. The most strongly down-regulated gene in periodontitis was the LPS biosynthesis protein WbpG. The adhesin FadA was strongly expressed both in laboratory culture and *in vivo* (**Supplementary Table S1 Sheet 10** and **Table 6**).

**Table 5:** Differentially expressed genes (15 most up- and down-regulated in periodontal pocket) between periodontitis and laboratory culture in *Fusobacterium nucleatum.*

Genes	log2FC	log2CPM	FDR	Product
992844	8.017376	9.386693	1.02E-06	Hemin receptor
991530	7.958785	10.18326	3.03E-05	Cytochrome C-type biogenesis protein CcdA
992301	6.994423	9.333086	2.66E-08	Sodium-dependent tyrosine transporter
993078	6.637803	10.87725	6.30E-05	Thiol:disulfide interchange protein TlpA
992331	6.282339	12.31312	8.09E-10	Acyl-CoA dehydrogenase
992475	6.080619	10.48263	2.66E-08	168 kDa surface-layer protein
993221	5.992068	7.534582	2.01E-07	Na(+)-linked D-alanine glycine permease
992567	5.841546	8.935339	0.000108	ABC transporter
992565	5.693574	8.578332	0.000561	ABC transporter
992642	5.658047	4.802479	9.15E-05	Pyruvate-flavodoxin oxidoreductase
993083	5.352238	12.57303	0.001161	Bifunctional methionine sulfoxide reductase A/B protein
992564	5.321549	7.156876	0.001161	Flavodoxin
992833	5.064472	1.627067	0.022785	Crp/Fnr family transcriptional regulator
992566	5.046786	4.002014	0.006948	Cytoplasmic protein
993227	-12.2841	5.180294	0.002829	Glucosamine–fructose-6-phosphate aminotransferase
991497	-12.5217	5.210201	0.000697	DNA-dependent DNA polymerase III subunit alpha
991244	-12.6182	5.220475	0.000149	ABC transporter ATP-binding protein
992659	-13.7519	6.117626	2.87E-05	RNA-directed DNA polymerase
992085	-14.0511	6.357712	1.26E-05	MunI regulatory protein
991246	-14.9509	7.183505	0.000375	Heteropolysaccharide repeat-containing protein
991329	-15.0455	7.223317	4.88E-05	UDP-*N*-acetyl-D-quinovosamine 4-epimerase
992734	-15.1044	7.236464	1.09E-06	Quinovosaminephosphotransferae
992744	-15.1989	7.412729	0.00056	Glycosyl transferase
991554	-15.2229	7.479123	0.001161	Acetyltransferase
991291	-15.2735	7.360318	3.18E-07	Cysteine desulfurase NifS
992796	-15.848	7.846506	3.57E-08	Spore coat polysaccharide biosynthesis protein SpsF
992371	-15.8759	7.966607	0.000219	DNA helicase
991799	-16.1556	8.205795	0.000185	dTDP-4-dehydrorhamnose reductase
991238	-18.7406	10.56156	6.06E-05	LPS biosynthesis protein WbpG

**Table 6:** Highly expressed genes (log2CPM ≥ 12) in both periodontitis and laboratory culture in *F. nucleatum.*

Genes	log2FC	log2CPM	FDR	Product
992523	0.872939	15.99842	0.471227	Major outer membrane protein
992669	-0.05416	14.07705	0.976413	Elongation factor Tu
992711	1.770125	14.02654	0.059115	Protein translation elongation factor G
991879	-1.22912	13.85202	0.335407	NAD-specific glutamate dehydrogenase
991927	-1.40289	13.71499	0.057432	Pyruvate-flavodoxin oxidoreductase
993140	-0.01272	13.14182	1	Adhesin fadA
992573	-0.89513	13.05281	0.389573	Flavodoxin FldA
991590	-0.10646	12.99426	0.953853	Acyl-CoA dehydrogenase
992883	1.998293	12.88908	0.062815	50S ribosomal protein L2
992123	0.779968	12.87944	0.536467	DNA-directed RNA polymerase subunit alpha
992432	-0.46743	12.73727	0.681545	Electron transfer flavoprotein subunit alpha
993261	-1.46781	12.46495	0.137923	Acyl-CoA dehydrogenase
991602	0.252386	12.40325	0.813884	Formate acetyltransferase
992665	1.086605	12.3774	0.266361	Preprotein translocase subunit SecY
991878	-0.11273	12.27671	0.951961	DNA-binding protein HU
993122	-1.3506	12.16025	0.130682	Urocanate hydratase
992880	1.638034	12.01259	0.071342	50S ribosomal protein L4

### Microdiversity of Gene Expression in the Periodontal Community

To investigate the intra-species diversity of these three species in the periodontal communities, we identified SNPs (single nucleotide polymorphism) by performing variants calling analysis. For *P. gingivalis* we detected 29 variants of transcripts when matching the reads to the genome in single laboratory culture. However, 23,783 variants were discovered in the communities from patients with periodontitis. Only 16 variants were shared by both conditions (**Supplementary Table S1 Sheet 15**). Similar findings were observed for *T. denticola*. In laboratory culture, 314 variants were detected, whereas 62,145 variants were found in communities from patients with periodontitis, and only one was shared between both conditions (**Supplementary Table S1 Sheet 15**). The CRISPR-Cas genes and ABC transporters were relatively more variable in *P. gingivalis* and *T. denticola* than the other genes (**Supplementary Table S1 Sheets 16, 17**).

**FIGURE 4:**
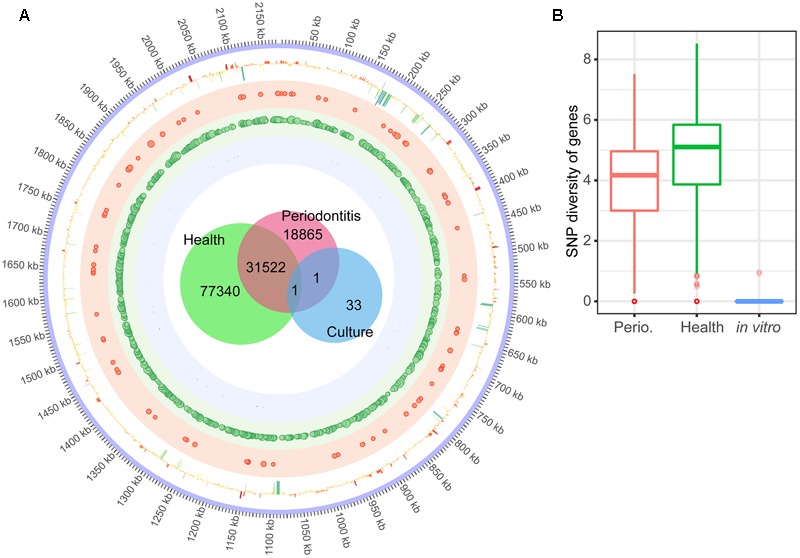
Single Nucleotide Polymorphisms (SNPs) in the transcriptome of *F. nucleatum* in the periodontal niche and in laboratory culture. **(A)** The innermost Venn diagram shows the number of SNPs of *F. nucleatum* transcripts in the periodontal community of individuals with periodontitis, without periodontitis, and laboratory culture. The circle shows the SNPs mapped to the genome of *F. nucleatum.* Size of the dots in the shaded red, green and blue circles illustrates the frequency (>50) of variants in a 1 kb window of *F. nucleatum* in periodontitis, health and laboratory culture, respectively. The outermost circle shows the log2FC of each gene in periodontitis compared to laboratory culture. **(B)** Frequency of SNPs per gene calculated as Shannon diversity (see Section “Materials and Methods” for details). The median, inter quantile range and outliers are shown in the boxplot.

For *F. nucleatum*, the large number of transcripts allowed to map SNPs to the genome (**Figure 4A**). In health, the complete genome was covered by SNPs. A total of 127,729 SNPS were discovered *in vivo*, while only 35 were found in laboratory culture and a mere two shared between both conditions. **Figure 4B** shows variant diversity calculated as Shannon index of diversity per gene. This value was slightly larger in health, but this was due to the larger number of samples from healthy individuals (8 compared to 4). Iron uptake related genes of *F. nucleatum* were more variable in periodontitis (**Supplementary Table S1 Sheet 18**). The number of SNPs was correlated with the coverage of the gene in question, so that the genes with higher coverage tended to hold more variants for all three species (**Supplementary Table S1 Sheets 16–18**).

## Discussion

The data clearly show that gene expression of all three bacterial pathogens studied here was fundamentally different in periodontitis (*in vivo)* from that found on laboratory media. Among the factors that can be expected to shape *in vivo* gene expression are the type of carbon source used, the availability of micronutrients like iron and vitamins, competition, cross-feeding and co-aggregation with other bacterial species present in the periodontal microbiota, phages, and interactions with the human host. Examples for each of these influences on *in vivo* gene expression were found in the data presented here. We will discuss the most striking findings below.

For all three pathogens, more genes were down- than up-regulated in the natural niche. This may be because the periodontal community is a more competitive environment and it is not as nutrient rich as an optimized culture medium. This was most pronounced in *T. denticola* and *F. nucleatum.* Of the total number of 987 differentially expressed genes of *T. denticola*, 74% were down-regulated, and from the 493 differentially expressed genes of *F. nucleatum* in the periodontal pockets from individuals with periodontitis compared to laboratory culture, 71% were down-regulated *in vivo*. By contrast, only 291 genes significantly changed their gene expression in *P. gingivalis*, and of those 57% were down-regulated *in vivo*. The laboratory data for *P. gingivalis* covered a broad physiological range, since they were derived from three different cultivation media, of which two were complex media and one was a chemically defined minimal medium (Hovik et al., 2012). The pair-wise differences in gene expression between these media were 218, 223, and 267 genes in total at a level of more than 1.5-fold change in mean RNA-seq read count (Hovik et al., 2012), thus these differences were much smaller than those observed here between *in vivo* and laboratory conditions which were in total 784 differentially expressed genes with at least 2.46-fold changes. We conclude that the natural niche has a much stronger influence on gene expression of a pathogen than the chosen cultivation medium, and that *in vivo* only a small subset of its genes are actually highly expressed, which may reflect distinct selective pressure which is not present during mono-culture cultivation on a laboratory medium.

To get an overview of the functions that were differentially expressed *in vivo* we analyzed GO term enrichment. Some similarities between the three pathogens were observed, i.e., GO terms for protein metabolism, translation, cell adhesion, iron transporters, and pathogenesis were up-regulated in at least two of them, indicating that the cells were highly active *in vivo* and most probably more pathogenic than on laboratory media.

We then inspected those genes that were highly expressed both *in vivo* and in the laboratory, and those that were differentially expressed. For *P. gingivalis* we observed consistent expression of several of its virulence factors under all conditions, namely the hemagglutinin proteins HagA and HagE, the receptor antigen protein RagA, and the arginin-specific cystein proteinase (gingipain). Among the genes up-regulated *in vivo* were a fimbrilin gene and two cystein proteinase genes, required for adhering to other bacterial species and host tissue (Nelson et al., 2003). The most strongly up-regulated genes (average fold change of 70) belonged to the *hmu* hemin/heme uptake locus (PG1551-PG1556, *hmuY, hmuR, hmuS, hmuT, hmuU, hmuV*) which has been shown to be important for virulence in an animal model (Kesavalu et al., 2003; Lewis et al., 2006). Here we confirm that it is indeed the major route to obtain iron for *P. gingivalis* in the human periodontal pocket. The up-regulation of ABC transporters (Schneider and Hunke, 1998) and TonB-dependent receptors (Noinaj et al., 2010) *in vivo* suggests competition for nutrient uptake. Strikingly CRISPR-Cas3 as well as restriction-modification enzymes, transposases, integrases and helicases were down-regulated *in vivo*. Since phage attack, extracellular DNA, and stress conditions requiring DNA re-arrangement should be rather more frequent under *in vivo* conditions than in the laboratory, the contrary would have been expected. The most strongly down-regulated gene was *pgaA* enoding a surface protein.

*Treponema denticola* showed the most dramatic differences between gene expression *in vivo* and in laboratory mono-culture, possibly reflecting the fact that this spirochaete invades host cells which is not mimicked in culture. 730 genes were significantly down-regulated in the periodontal niche, covering all aspects of bacterial physiology, including, as in *P. gingivalis*, CRISPR-related genes, DNA-re-organization, and phage related genes, but also many other metabolic functions, including lipoproteins, proteases, cobalamin-biosynthesis, and many more. The most strongly differentially expressed gene was hemolysin which was down-regulated (log2FC = 16.251) in the periodontal pocket indicating that *T. denticola* does not obtain its iron by lysing erythrocytes *in vivo*. By contrast, it uptakes iron directly from the environment using specific iron transporters and ABC transporters, which were the most strongly up-regulated genes *in vivo*. Under both, *in vivo* conditions and laboratory culture, the most highly expressed genes of *T. denticola* were flagella filament proteins, the Msp protein that interacts with the immune system, glycine metabolism enzymes, and dentilisin, a surface protease (Dashper et al., 2011) that can degrade interleukins (Miyamoto et al., 2006) and can bind to *P. gingivalis* fimbriae (Hashimoto et al., 2003). The fimbrilin gene of *P. gingivalis* was strongly up-regulated in the periodontal pocket (see above). Our data thus confirm that those two red-complex pathogens can indeed co-aggregate *in vivo* so that the non-motile *P. gingivalis* may take advantage from binding to the motile spirochaete.

*Fusobacterium nucleatum* is of tremendous medical importance, yet it is relatively understudied, possibly because of its obligate anaerobic lifestyle which requires specialized equipment for culturing. The most strongly up-regulated gene in the periodontal pocket was a hemin receptor, thus *F. nucleatum* obtains its iron from hemin *in vivo* like *T. denticola* and *P. gingivalis*. Genes encoding enzymes of the primary metabolism were down-regulated in the periodontal pocket, confirming that laboratory culture media offer more substrates than are actually utilized *in vivo*. The most important virulence factor of *F. nucleatum*, the adhesin Fad (Rubinstein et al., 2013), was highly expressed in the periodontal pocket in health and disease as well as in laboratory mono-culture. It can therefore be expected to be a reliable biomarker for *F. nucleatum* pathogenicity.

**FIGURE 5:**
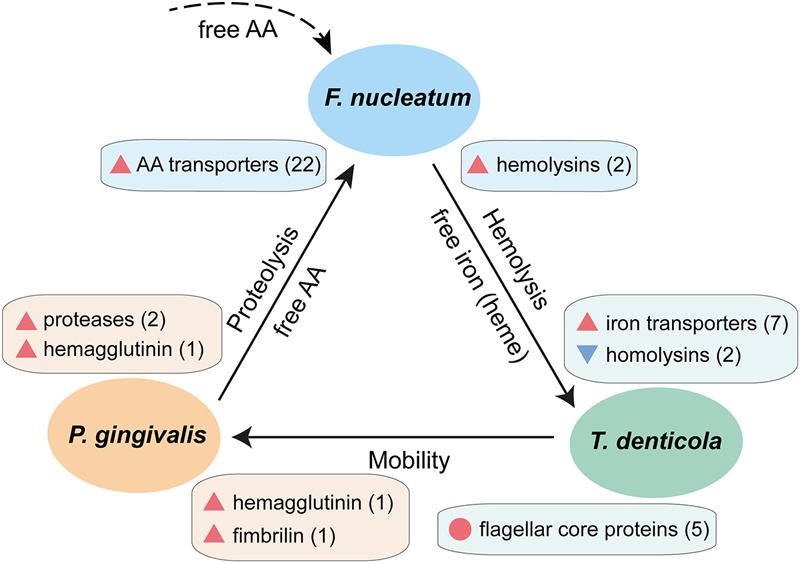
Schematic interaction model showing synergistic pathogenicity of *F. nucleatum, P. gingivalis*, and *T. denticola* based on their transcriptional profiles in chronic periodontitis. Blue triangles denote down-regulation, red triangles up-regulation *in vivo*, the number in parentheses indicate the number of genes of this particular function, and the red circle denotes a set of genes which are highly expressed both *in vivo* and in laboratory culture. The dashed line indicates contribution of free amino acids by other members of the periodontal microbiota.

Recent animal studies indicate that co-infection with *P. gingivalis, T. denticola*, and *F. nucleatum* can significantly enhance tissue damage during periodontitis compared with mono-infection with these species (Kesavalu et al., 2007; Polak et al., 2009; Dashper et al., 2011). In our data, we found clues for their synergistic pathogenicity. Based on the transcriptional profiles of these key members of the periodontal community *in vivo*, a schematic interaction model can be established which shows two-way synergistic interactions, i.e., each species gives and takes (**Figure 5**). The upregulation of many surface proteins *in vivo* suggests enhanced adhesion in the periodontal niche (Lee et al., 2005). The data suggest that *P. gingivalis* attached to *T. denticola* or other bacteria via hemagglutinin and fimbriae to improve its mobility (**Figure 5**). As *F. nucleatum* exhibits no or weak intrinsic proteolytic activity, it will profit from the coexistence with other species with strong proteolytic activities such as *P. gingivalis* (Bolstad et al., 1996). Shah and co-workers observed that *F. nucleatum* preferentially uses peptides instead of free amino acids (Shammas et al., 1993). Here we found that *P. gingivalis* and *T. denticola* up-regulated cysteine proteases while *F. nucleatum* massively up-regulated peptide transporters, thus it could take advantage of the proteolytic activity of other species especially *P. gingivalis*. A distinct form of shared labor was observed for iron, one of the key elements in pathogenicity (Skaar, 2010). *F. nucleatum* engaged in lysis of erythrocytes by strong upregulation of hemolysins; accordingly, *T. denticola* down-regulated hemolysins. Both *T. denticola* and *P. gingivalis* likely obtained their iron by binding heme or uptaking free iron, thus they profited from the hemolytic activity of *F. nucleatum*.

The *in vivo* metatranscriptomes showed an unexpectedly high level of microdiversity for all three pathogens. We observed 23,783 single nucleotide variants (SNPs) for *P. gingivalis*, 62,145 for *T. denticola* and 127,729 for *F. nucleatum* transcripts. In *F. nucleatum*, SNPs were found in all chromosomal genes. The number of SNPs increased with the number of periodontal samples analyzed, and it tended to be higher for highly expressed genes. In an attempt to compare the number of SNPs per gene in health and periodontitis, we calculated the Shannon Index of diversity per gene. It was slightly higher in health, reflecting the larger number of samples available in health.

The data show that microdiversity is a common trait of pathogens in the periodontal pocket and affects every gene. Therefore, it likely reflects continuous micro-evolution *in vivo*. The average age of the subjects in our study was 52 (health) and 56 years (chronic periodontitis) so the microbial communities had co-evolved for a long time. Such SNPs could occur through the mutagenic impact of DNA damaging agents like reactive oxygen species, antibiotics or toxins, but also through horizontal gene transfer (HGT). Dental plaque is a hot spot of HGT due to the close physical contact between the microorganisms and their fast growth. *S. mutans*, for example, is genetically competent in a density dependent fashion and has integrated the regulation of competence and the synthesis of mutacins through the alternative sigma factor sigX (Reck et al., 2015).

Such microdiversity has been observed in metagenomics studies of various habitats before (Larkin and Martiny, 2017) resulting in the concept of “ecotypes” (Farrant et al., 2016). The functional importance of population microdiversity could be demonstrated for the colonization of premature babies, where polymorphism of *Citrobacter koseri* genomes at 47 sites was found and it could actually be shown that a specific subpopulation was restricted to the gut (Olm et al., 2017). The adaptive importance of microdiversity in the periodontal pocket remains to be explored.

## Conclusion

In the natural environment of the periodontal niche, bacteria need to fight for their survival due to the shortage of essential nutrients and clearance of the host immune system. Under such conditions, bacteria upregulate the genes which they need the most and cooperate with each other to improve their fitness. By comparing the transcriptional profiles from *in vivo* conditions to gene expression in single culture, we were able to identify those genes which help them to obtain essential nutrients, evade the immune system and cooperate. When we analyzed transcriptional profiles on different culture media for *P. gingivalis*, we found that there were differences but they were very small in comparison to *in vivo* expression.

Interfering with the genes upregulated by key microbial members (such as cysteine proteases and heme binding proteins of *P. gingivalis*, peptide transporters of *F. nucleatum*, iron transporters of *T. denticola*) may influence their abundance in the community and help to shift the dysbiosis toward eubiosis. Therefore, the findings in this study may provide insights for the development of novel therapeutic strategies specifically reducing certain key-stone pathogens and bringing back the community to an ecological equilibrium, rather than wiping it out completely using standard antibiotics.

## Author Contributions

HS performed the optimization of culture conditions for growth, the RNA extraction, quality checking, and mRNA enrichment. SB and MJ prepared the cDNA libraries and performed the Illumina sequencing. Z-LD designed the data analysis pipeline and analyzed the data. IW-D supported and supervised the research. Z-LD and IW-D wrote the manuscript, all authors reviewed the manuscript.

## Conflict of Interest Statement

The authors declare that the research was conducted in the absence of any commercial or financial relationships that could be construed as a potential conflict of interest.

## Footnotes

1 http://www.dsmz.de

## Supplementary Material

FIGURE S1 Relative abundance of transcripts from *Porphyromonas gingivalis, Treponema denticola*, and *Fusobacterium nucleatum* in the periodontal pocket metatranscriptomes. The red dots on the top of the bar indicate the communities in periodontitis.

TABLE S1 Legend for each sheet.

SHEET 1 The number of reads for *P. gingivalis, T. denticola* and *F. nucleatum* from laboratory culture.

SHEET 2 The number of reads for *P. gingivalis, T. denticola* and *F. nucleatum* from periodontal metatranscriptome.

SHEET 3 The gene expression of *P. gingivalis* in periodontal and mono-culture.

SHEET 4 The differential expression of *P. gingivalis* in periodontal niche vs. mono-culture.

SHEET 5 Gene ontology (GO) term enrichment analysis of *P. gingivalis*.

SHEET 6 The gene expression of *T. denticola* in periodontal and mono-culture.

SHEET 7 The differential expression of *T. denticola* in periodontal niche vs. mono-culture.

SHEET 8 Gene ontology term enrichment analysis of *T. denticola*.

SHEET 9 The gene expression of *F. nucleatum* in periodontal niche and mono-culture.

SHEET 10 The differential expression of *F. nucleatum* in periodontitis vs. mono-culture.

SHEET 11 The differential expression of *F. nucleatum* in health vs. mono-culture.

SHEET 12 The differential expression of *F. nucleatum* in periodontitis vs. health.

SHEET 13 Gene ontology term enrichment analysis of *F. nucleatum* in periodontitis compared to mono-culture.

SHEET 14 Gene ontology term enrichment analysis of *F. nucleatum* in periodontitis compared to health.

SHEET 15 The number of variants detected in the transcriptomes of *P. gingivalis, T. denticola*, and *F. nucleatum* from periodontal niche and laboratory culture.

SHEET 16 The variants per gene in the transcriptome of *P. gingivalis*.

SHEET 17 The variants per gene in the transcriptome of *T. denticola*.

SHEET 18 The variants per gene in the transcriptome of *F. nucleatum*.
