# Supplementary figures and images for: Worlds Apart – Transcriptome Profiles of Key Oral Microbes in the Periodontal Pocket Compared to Single Laboratory Culture Reflect Synergistic Interactions
Source: Front Microbiol. 2018 Feb 6;9:124. doi: 10.3389/fmicb.2018.00124 (PMC5807917; doi:10.3389/fmicb.2018.00124)

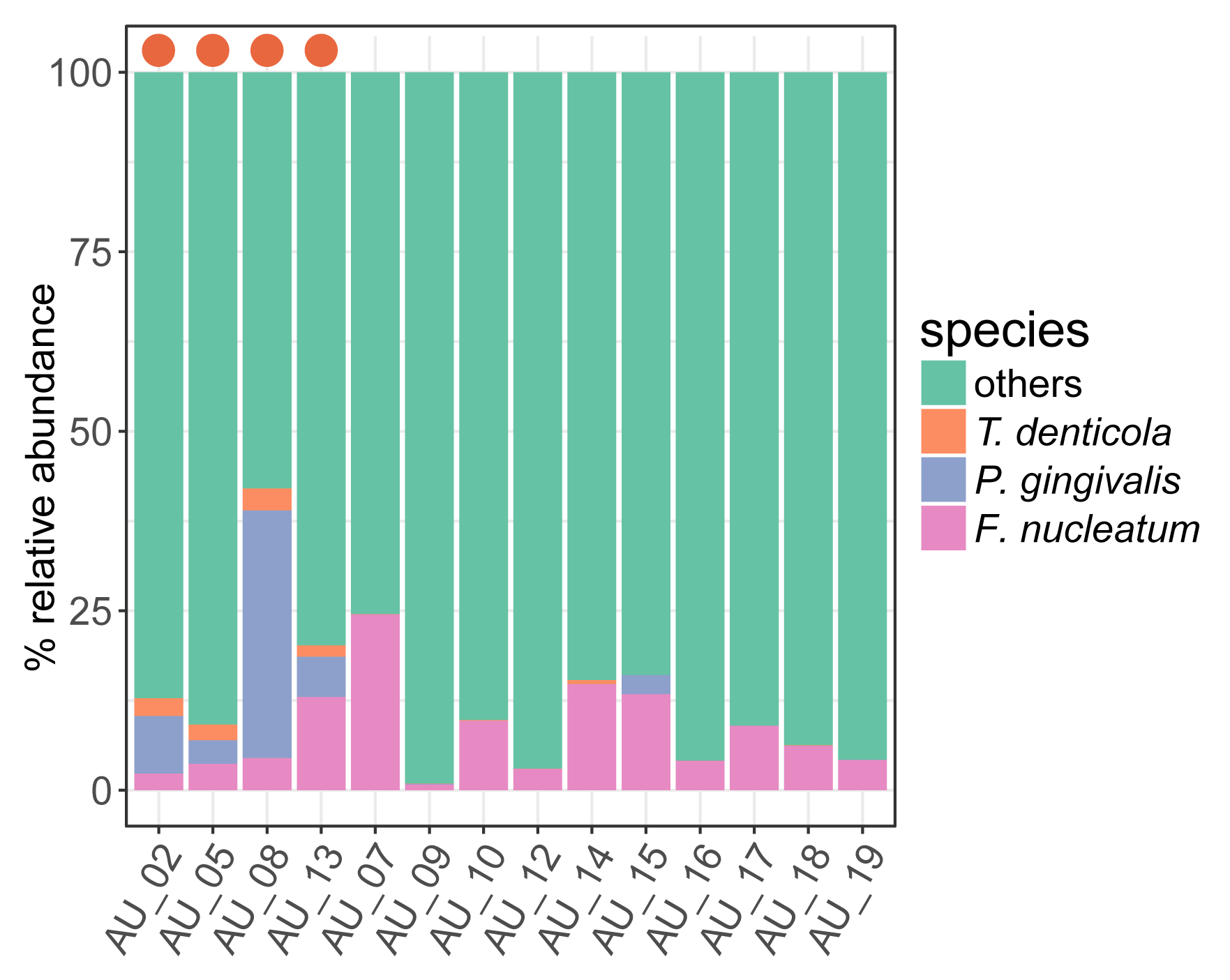

Supplement: FIGURE S1 — Relative abundance of transcripts from Porphyromonas gingivalis, Treponema denticola, and Fusobacterium nucleatum in the periodontal pocket metatranscriptomes. The red dots on the top of the bar indicate the communities in periodontitis. [file Image_1.TIF]
